# The association between haemoglobin levels in the first 20 weeks of pregnancy and pregnancy outcomes

**DOI:** 10.1371/journal.pone.0225123

**Published:** 2019-11-13

**Authors:** Deborah A. Randall, Jillian A. Patterson, Felicity Gallimore, Jonathan M. Morris, Therese M. McGee, Jane B. Ford, for the Obstetric Transfusion Steering Group

**Affiliations:** 1 The University of Sydney Northern Clinical School, Women and Babies Research, St Leonards, New South Wales, Australia; 2 Northern Sydney Local Health District, Kolling Institute, New South Wales, Australia; 3 Women’s and Newborn Health, Westmead Hospital, Westmead NSW, Australia; 4 The University of Sydney, Sydney NSW, Australia; university of campus biomedico, ITALY

## Abstract

**Background:**

Low haemoglobin has been linked to adverse pregnancy outcomes. Our study aimed to assess the association of haemoglobin (Hb) in the first 20 weeks of pregnancy, and restoration of low Hb levels, with pregnancy outcomes in Australia.

**Methods:**

Clinical data for singleton pregnancies from two tertiary public hospitals in New South Wales were extracted for 2011–2015. The relationship between the lowest Hb result in the first 20 weeks of pregnancy and adverse outcomes was determined using adjusted Poisson regression. Those with Hb <110 g/L were classified into ‘restored’ and ‘not restored’ based on Hb results from 21 weeks onwards, and risk of adverse outcomes explored with adjusted Poisson regression.

**Results:**

Of 31,906 singleton pregnancies, 4.0% had Hb <110 and 10.2% had ≥140 g/L at ≤20 weeks. Women with low Hb had significantly higher risks of postpartum haemorrhage, transfusion, preterm birth, very low birthweight, and having a baby transferred to higher care or stillbirth. High Hb was also associated with higher risks of preterm, very low birthweight, and transfer to higher care/stillbirth. Transfusion was the only outcome where risk decreased with increasing Hb. Risk of transfusion was significantly lower in the ‘restored’ group compared with the ‘not restored’ group (OR 0.39, 95% CI 0.22–0.70), but restoration of Hb did not significantly affect the other outcomes measured.

**Conclusions:**

Women with both low and high Hb in the first 20 weeks of pregnancy had higher risks of adverse outcomes than those with normal Hb. Restoring Hb after 20 weeks did not improve most adverse outcome rates but did reduce risk of transfusion.

## Introduction

One in 10 women suffer excessive bleeding after childbirth and 15% of these women will have a red blood cell transfusion.[1] An increasing rate of blood transfusion in the maternity population has been noted and is of concern.[2, 3] Pre-natal and antenatal detection and correction of anaemia may be an effective strategy to reduce the impact of blood loss following birth and improve birth outcomes.[4]

Iron requirements increase during pregnancy, mainly to expand red blood cell mass, fulfil the iron requirements of the fetus, and to compensate for blood loss at delivery.[5] While there is an increase in red blood cell volume during pregnancy, there is a larger increase in plasma volume, and this differential increase results in dilution of haemoglobin (Hb) in the blood during pregnancy.[6] In Australia, there is no agreed normal range for Hb concentration in pregnant women[7] and no existing population health data prospectively collecting Hb levels to examine ranges and their impact on outcomes. The World Health Organization (WHO) considers pregnant women with <110 g/L Hb as anaemic,[8] however, the cut-offs are derived predominantly from developing countries and are not necessarily generalisable.[7] The cut-offs recommended by the Centers for Disease Control and Prevention in the United States were developed using four European studies of healthy pregnant women receiving iron supplements.[9] They determined the following maximum Hb levels for diagnosing anaemia: 110 g/L in the first trimester, 105 g/L in the second trimester and 110 g/L in the third trimester.[9] No studies to date have been based on an Australian pregnant population.

Anaemia at the birth admission has been associated with higher caesarean section rates and adverse outcomes such as higher rates of postpartum haemorrhage (PPH), blood transfusion, and infant transfer to neonatal intensive care.[10] Anaemia in the first and second trimester of pregnancy has been associated with low birthweight and preterm birth,[11, 12] as have high Hb levels.[13]

The National Pregnancy Care Guidelines recommend all women have their Hb level checked at the first antenatal visit and again at approximately 28 weeks’ gestation.[14] While it is recommended that any anaemia be investigated and treated, routine iron supplementation is not recommended for every pregnancy.[15] Patient Blood Management guidelines aim to reduce obstetric blood transfusions by taking an individualised approach that attempts to reduce the need for transfusion and therefore avoid unnecessary exposure to blood and blood products.[16] Given the potential importance of pre-delivery iron status in helping women cope with blood loss associated with childbirth, it is important to understand the impact of low Hb early in pregnancy on adverse outcomes. At this point, it may be possible to restore the Hb levels. Our study therefore aimed to assess levels of Hb at ≤20 weeks and associations with PPH and blood transfusion at birth or postnatally, and whether restoration of Hb levels reduced the likelihood of PPH or transfusion and/or improved pregnancy outcomes.

## Materials and methods

### Design and setting

The study was a retrospective cohort study using hospital data, and was conducted in two large tertiary public hospitals in New South Wales, Australia, the Royal North Shore Hospital and Westmead Hospital. Singleton pregnancies in a five-year period from 1 January 2011 to 31 December 2015 were included.

### Data sources

Detailed data on maternal characteristics, pregnancy history, and birth factors were obtained from the ObstetriX database (‘birth data’), a clinical database that is completed by midwives at booking, at antenatal visits and at the birth admission, recording information for births of at least 20 weeks’ gestation or 400g birth weight. Data on age of the mother, country of birth (COB), inpatient and outpatient encounters for the perinatal period (from the start of pregnancy to six weeks postnatally) for the mother were obtained from the Electronic Medical Record (‘eMR’). Most diagnoses and procedures were coded according to the International Statistical Classification of Diseases and Related Health Problems, Australian Modification (ICD10-AM), and the Australian Classification of Health Interventions,[17] with a small minority coded using the SNOMED classification.[18] Hb results and test date were obtained from either the ObstetriX database, where they were manually entered, or eMR Pathology data (where results were from an onsite hospital pathology laboratory, or an off-site linked laboratory for Westmead only).

### Variables

To identify women with low Hb levels, all Hb results obtained in the first 20 weeks of the pregnancy were searched, and the lowest Hb result was classified into 10 g/L categories (<90, 90–99, 100–109 etc to 150–159 g/L), as well as three broad categories of <110 (range 45–109), 110–139 and 140+ (range 140–159) g/L. Hb results between 8 and 14 (n = 24) were re-coded to 80–140 due to likely transcription errors. All other results outside of probable values (45 to 159 g/L), as determined by the clinical authors and comparisons with haematocrit levels, were set to missing. We chose the lowest Hb in the first 20 weeks as we were interested in the Hb before any treatment. Those without a Hb result in the first 20 weeks were excluded from the study, and the distribution of their characteristics compared against the distribution of those in the study using standardized percentage differences.[19] Women with a Hb result <110g/L at ≤20 weeks were further examined to determine their subsequent mean Hb across the remainder of the gestational period before birth (>20 weeks). These women were classified as ‘restored’ if their mean Hb after 20 weeks gestation was 110 g/L or more, ‘not restored’ if the mean Hb results were <110 g/L, and ‘no further results’ if no further Hb results were recorded after 20 weeks and before birth.

Main outcomes were postpartum haemorrhage (PPH; a combined indicator using birth and eMR data), and transfusion at the birth or 6 weeks postnatally (birth and eMR data). Further outcomes included preterm birth (<37 weeks gestation; birth data) divided into planned (induction or pre-labour caesarean) and spontaneous, stillbirth (birth data), neonatal transfer to special care nursery (SCN) or neonatal intensive care unit (NICU; birth data), small for gestational age <10% (SGA; birth data),[20] and very low birthweight (<1500g; birth data). See S1 Table for more details.

Maternal age, COB, and postcode of residence were obtained from the eMR. Parity, smoking, body mass index (BMI), gestational diabetes, pregnancy hypertension, abnormal placenta site, and previous history of anaemia, diabetes, hypertension and major uterine surgery, were obtained from the birth data. Socio-economic status was matched to the Index of Relative Socio-Economic Advantage and Disadvantage (IRSAD)[21] using postcode and divided into population quintiles. Additional birth factors that could be mediating causes of PPH or transfusion, i.e. labour onset, mode of birth, and perineal tears, were obtained from the birth data.

### Statistical methods

Characteristics of women in the three broad Hb groups (<110, 110–139, 140+ g/L) were compared using χ^2^ tests. Outcome data were also compared between these broad Hb groups, using percentages. The relative rates of PPH, transfusion, preterm birth, small for gestational age, very low birthweight and transfer to NICU/SCN or stillbirth, were investigated by more detailed Hb groups (<90, 90–99, 100–109, 110–119, 120–129, 130–139, 140–149, 150–159 g/L) using modified Poisson regression models, adjusting for maternal characteristics (age, BMI, country of birth, parity, SES quintile), pregnancy risk factors (smoking, gestational diabetes and hypertension, pre-existing diabetes and hypertension, previous uterine surgery, abnormal placenta site, antenatal haemorrhage), and potentially mediating factors (labour onset, mode of birth, perineal tears). The composite indicator of stillbirth or transfer to higher care was used, as there were not enough stillbirths to look at separately. The risk of these same outcomes for women with their Hb ‘restored’ and ‘not restored’ were also estimated using modified Poisson, and adjusted with a smaller set of covariates that differed across the Hb groups and also influenced the outcomes (country of birth, SES quintile, parity, smoking, BMI, labour onset, mode of birth, perineal tears). There were not enough stillbirths or babies with very low birth weights to examine separately, so these were combined in a composite neonatal adverse outcome indicator with transfer to higher care.

### Ethics

Ethical approval for this study was obtained from Northern Sydney Local Health District Ethics Committee (LNR/17/HAWKE/32).

## Results

**Fig 1:**
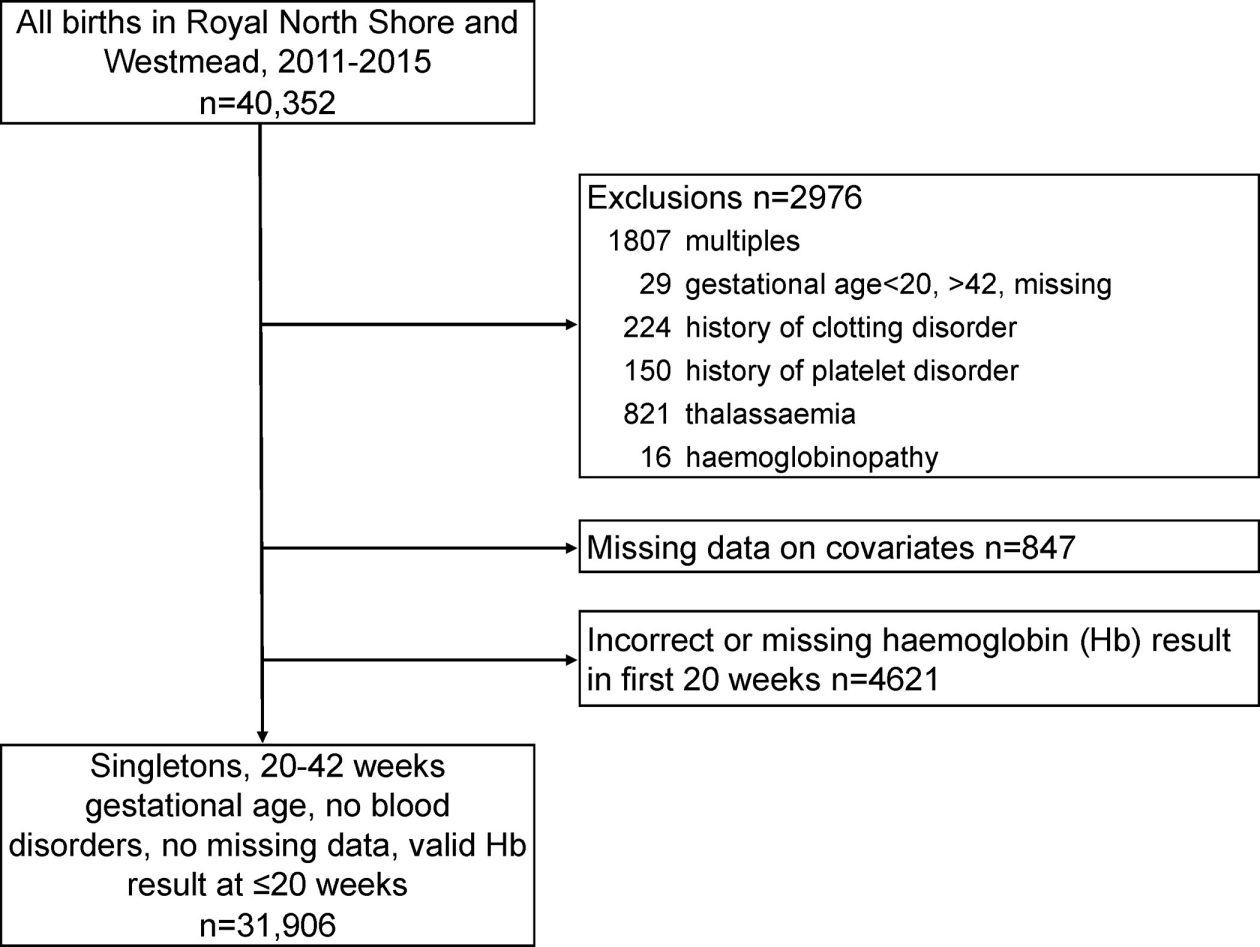
Study population.

There were 40,352 births at the Royal North Shore and Westmead hospitals between 1 January 2011 and 31 December 2015. After exclusions (see Fig 1), 31,906 singletons births remained. A number of women were removed from the analysis due to missing Hb in the first 20 weeks (n = 4621). A comparison of characteristics for those women missing Hb and the final analysis population is in S2 Table. Women missing a Hb result in the first 20 weeks of pregnancy were similar to those in the study population, but were less likely to have been born in Southern Asia, and more likely to have been born in Oceania, had 2 or more previous pregnancies, had a previous history of B12/folate deficiency, and have smoked during pregnancy (standardised difference >0.1).

The mean of the lowest Hb at ≤20 weeks recorded for each woman was 127.4 g/L (standard deviation 10.0) and the median was 128 (interquartile range 13). Overall, 4.0% of women had Hb <110 g/L, 85.7% had Hb 110–139 g/L, and 10.2% had Hb 140+ g/L at ≤20 weeks. Women with a Hb <110 g/L at ≤20 weeks were less likely than those with Hb 110–139 and 140+ to be born in Australia, Europe, South- and North-East Asia and the Americas, and were more likely to be born in Africa and the Middle East and Southern and Central Asia (Table 1). There was also a socio-economic gradient, with the likelihood of living in the most advantaged areas increasing with increasing Hb levels. Women with lower Hb had higher parity, and were more likely to have a BMI of <18.5 and have a history of iron-deficiency anaemia, than those with medium Hb, who in turn had higher rates than those with Hb 140+. The low Hb group were more likely to have planned caesareans, less likely to have unassisted vaginal births, and among vaginal births, there were similar rates of perineal trauma.

**Table 1:** Characteristics of the population by hospital of birth and haemoglobin (Hb) cut-points.

	Total		Lowest Hb in first 20 wks						
			<110 g/L		110–139 g/L		140+ g/L		χ2
	n = 31,906		n = 1282		n = 27,356		n = 3268		test
	Col % (n)		Col % (n)		Col % (n)		Col % (n)		
***Maternal characteristic***									
**Age group**‡									
<20	1.1%	(355)	1.6%	(21)	1.1%	(307)	0.8%	(27)	
20–34	76.4%	(24362)	78.0%	(1000)	76.2%	(20847)	77.0%	(2515)	
35+	22.5%	(7189)	20.4%	(261)	22.7%	(6202)	22.2%	(726)	
**Country of birth**‡									
Australia	35.2%	(11227)	25.0%	(321)	34.6%	(9457)	44.3%	(1449)	***
Oceania (rest)	3.6%	(1134)	3.7%	(48)	3.5%	(969)	3.6%	(117)	
Europe	6.8%	(2158)	3.5%	(45)	6.8%	(1867)	7.5%	(246)	
Africa and the Middle East	10.2%	(3253)	14.4%	(184)	10.4%	(2850)	6.7%	(219)	
South- and North-East Asia	19.8%	(6322)	13.9%	(178)	19.7%	(5391)	23.0%	(753)	
Southern Asia	19.8%	(6332)	33.6%	(431)	20.3%	(5543)	11.0%	(358)	
Central Asia	2.3%	(721)	4.6%	(59)	2.3%	(630)	1.0%	(32)	
Americas	2.4%	(759)	1.2%	(16)	2.4%	(649)	2.9%	(94)	
**Socio-economic status quintile**‡									
1—most disadvantaged	21.3%	(6811)	29.3%	(375)	21.4%	(5851)	17.9%	(585)	***
2	4.4%	(1406)	5.8%	(74)	4.3%	(1184)	4.5%	(148)	
3	22.6%	(7206)	29.3%	(376)	22.6%	(6176)	20.0%	(654)	
4	19.3%	(6147)	14.8%	(190)	19.3%	(5276)	20.8%	(681)	
5—most advantaged	32.4%	(10336)	20.8%	(267)	32.4%	(8869)	36.7%	(1200)	
***Previous history***									
**Parity**†									
No previous births	47.4%	(15117)	45.7%	(586)	46.5%	(12713)	55.6%	(1818)	***
1 previous birth	34.5%	(10999)	31.5%	(404)	35.0%	(9581)	31.0%	(1014)	
2+ previous births	18.1%	(5790)	22.8%	(292)	18.5%	(5062)	13.3%	(436)	
**Medical history**									
Pre-existing diabetes†	0.9%	(287)	2.0%	(25)	0.8%	(231)	0.9%	(31)	***
Previous gestational diabetes†	4.3%	(1358)	4.1%	(53)	4.3%	(1170)	4.1%	(135)	
Pre-existing hypertension	6.3%	(1997)	7.6%	(98)	6.0%	(1631)	8.2%	(268)	***
Previous major uterine surgery†	16.4%	(5229)	20.1%	(258)	16.5%	(4526)	13.6%	(445)	***
**Previous history of anaemia†**									
Iron-deficiency	14.9%	(4764)	44.4%	(569)	14.3%	(3921)	8.4%	(274)	***
B12/folate deficiency	0.3%	(97)	1.1%	(14)	0.3%	(74)	0.3%	(9)	***
***Current pregnancy risks***									
Smoked during pregnancy‡	2.8%	(892)	2.9%	(37)	2.6%	(715)	4.3%	(140)	***
Gestational diabetes‡	11.6%	(3688)	10.9%	(140)	11.4%	(3107)	13.5%	(441)	**
Pregnancy hypertension‡	5.0%	(1592)	5.1%	(66)	4.8%	(1306)	6.7%	(220)	***
Abnormal placenta site‡	1.4%	(437)	2.2%	(28)	1.3%	(366)	1.3%	(43)	*
Antepartum haemorrhage‡	3.9%	(1260)	5.3%	(68)	3.8%	(1044)	4.5%	(148)	**
**Body Mass Index**†									
<18.5	5.9%	(1879)	10.0%	(128)	5.8%	(1589)	5.0%	(162)	***
18.5–24.99	59.6%	(19023)	57.6%	(739)	60.0%	(16403)	57.6%	(1881)	
25+	34.5%	(11004)	32.4%	(415)	34.2%	(9364)	37.5%	(1225)	
***Birth factors***									
**Labour onset**									
Spontaneous	52.5%	(16739)	47.7%	(612)	52.6%	(14381)	53.4%	(1746)	***
Induction	30.6%	(9766)	30.8%	(395)	30.5%	(8338)	31.6%	(1033)	
Pre-labour caesarean	16.9%	(5401)	21.5%	(275)	17.0%	(4637)	15.0%	(489)	
**Mode of birth**									
Vaginal unassisted	57.2%	(18260)	52.6%	(674)	57.5%	(15736)	56.6%	(1850)	**
Vaginal instrumental–forceps	7.8%	(2480)	7.3%	(93)	7.8%	(2133)	7.8%	(254)	
Vaginal instrumental–vacuum	4.9%	(1571)	5.9%	(76)	4.8%	(1323)	5.3%	(172)	
Caesarean section	30.1%	(9595)	34.2%	(439)	29.8%	(8164)	30.4%	(992)	
**Perineal trauma (for vaginal births only n = 22,311)**									
None	29.1%	(6482)	33.3%	(281)	28.8%	(5536)	29.2%	(665)	
1^st^ degree / Other	30.3%	(6758)	26.8%	(226)	30.5%	(5846)	30.1%	(686)	
2^nd^ degree	36.1%	(8052)	35.0%	(295)	36.1%	(6931)	36.3%	(826)	
3^rd^ or 4th degree	4.6%	(1019)	4.9%	(41)	4.6%	(879)	4.3%	(99)	

† Recorded at booking

‡ Recorded at birth admission

χ^2^ test significance

*p<0.05

**p<0.01

***p<0.001

The most common adverse pregnancy outcomes were infant transferred to higher care (16.0%), PPH (13.6%), a small for gestational age infant (9.5%), and preterm birth (7.1%) (Table 2). Those with Hb <110 g/L had a higher percentage of all adverse outcomes, compared to the group with Hb 110–139 g/L. Those with Hb 140+ g/L were slightly more likely to have many of the adverse outcomes than the normal Hb group, but were less likely than those with lower Hb to have a transfusion.

**Table 2:** Outcomes by lowest haemoglobin (Hb) at 20 weeks gestation or less.

	Total		Lowest Hb in first 20 wks						
			<110 g/L		110–139 g/L		140+ g/L		χ2
	n = 31,906		n = 1282		n = 27,356		n = 3268		test
	Col % (n)		Col % (n)		Col % (n)		Col % (n)		
Adverse maternal outcomes									
Postpartum haemorrhage	13.6%	(4355)	15.5%	(199)	13.4%	(3674)	14.7%	(482)	*
Transfusion	2.1%	(659)	4.9%	(63)	2.0%	(542)	1.7%	(54)	***
Preterm birth (<37 wks)	7.1%	(2281)	10.8%	(138)	6.8%	(1848)	9.0%	(295)	***
Planned preterm	3.7%	(1165)	6.7%	(86)	3.4%	(936)	4.4%	(143)	
Spontaneous preterm	3.5%	(1116)	4.1%	(52)	3.3%	(912)	4.7%	(152)	
Adverse neonatal outcomes									
Stillbirth	0.7%	(235)	2.0%	(25)	0.7%	(186)	0.7%	(24)	***
Small for gestational age	9.5%	(3031)	12.1%	(155)	9.4%	(2582)	9.0%	(294)	**
Very low birthweight (<1500g)	2.0%	(638)	3.7%	(47)	1.9%	(512)	2.4%	(79)	***
Transfer to NICU/SCN	16.0%	(5112)	19.7%	(253)	15.6%	(4272)	18.0%	(587)	***

χ^2^ test significance:

*p<0.05

**p<0.01

***p<0.001

**Fig 2:**
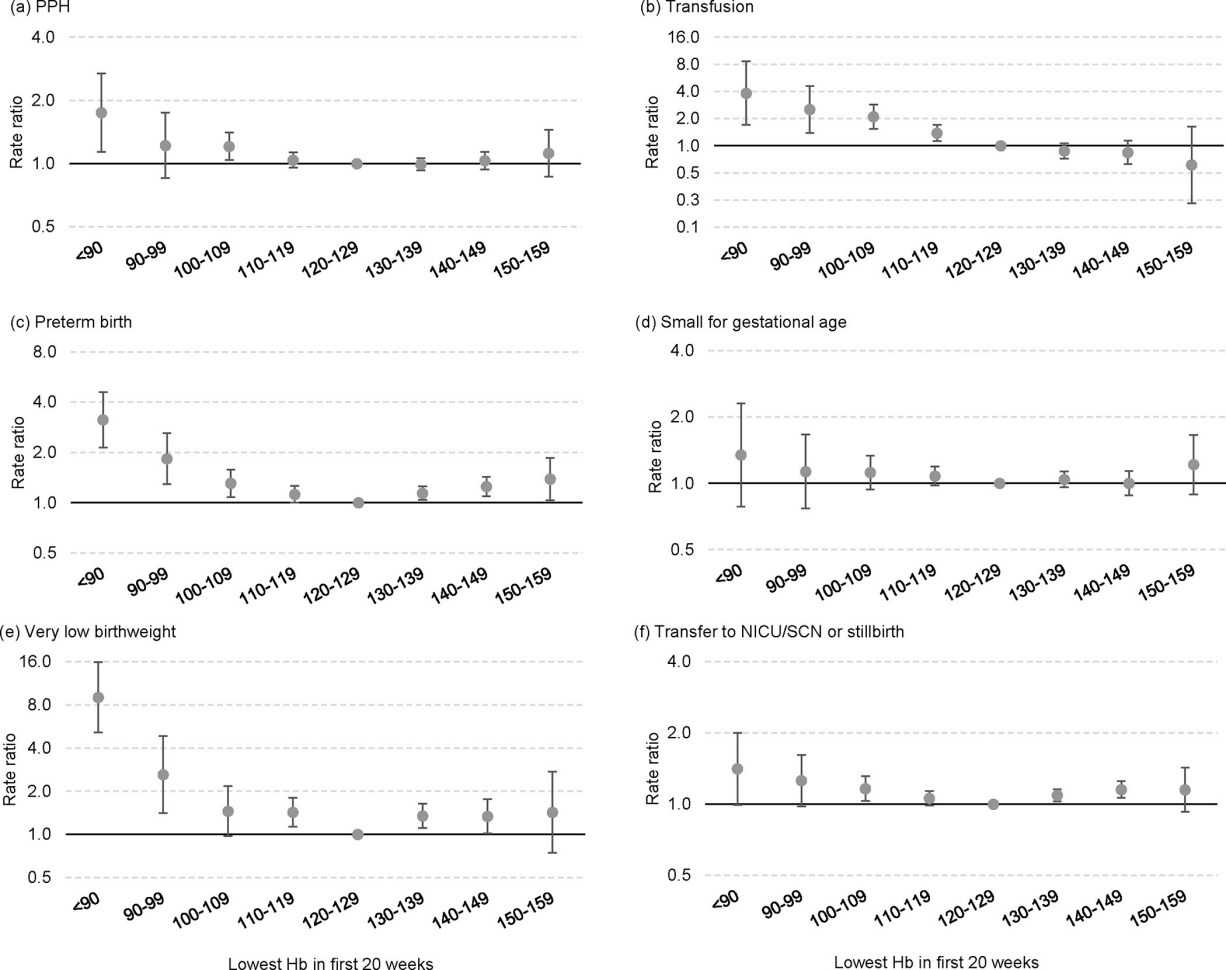
Adjusted rate ratios for individual outcomes by lowest haemoglobin (Hb) at 20 weeks gestation or less.

Fig 2 shows the adjusted rate ratios for adverse outcomes by the detailed Hb groups. There was a U-shaped relationship between Hb and all of the adverse outcomes except for transfusion, after adjusting for covariates in multivariable models. For most outcomes, the risk was comparatively higher at the lower Hb levels than the higher ones, with the risk starting to increase at Hb levels less than 120 (compared with 120–129 g/L). The U-shaped relationship was only very slight for PPH, with the adjusted rates among those with the highest Hb (150–159) only 1.1 and not significant. There was a linear relationship between Hb and transfusion rate, with the transfusion rate significantly higher in the Hb groups <120 g/L, and lower in the Hb groups ≥130 g/L (although not reaching significance) compared with 120–129 g/L group.

Of 1282 women with Hb <110 g/L at ≤20 weeks, 38% (n = 492) had a mean Hb from 21 weeks to birth of ≥110g/L (‘restored’), 38% (n = 488) did not (‘not restored’), and an additional 24% (n = 302) did not have any Hb results from 21 weeks onwards (Table 3). We compared the risk of adverse outcomes by ‘restored’ and ‘not restored’ Hb groups, adjusting for covariates. The adjusted risk of transfusion was significantly lower in the ‘restored’ group compared with the ‘not restored’ group, but there was no significant increased or decreased risk for the other outcomes.

**Table 3:** Outcomes among those with low Hb at 20 weeks gestation or less, by Hb status in the remaining weeks.

	Total		Hb status in remaining pregnancy						Restored versus not restored
			No further Hb		Restored		Not restored		
	n = 1282		n = 281		n = 481		n = 472		
	Col % (n)		Col % (n)		Col % (n)		Col % (n)		aRR (95% CI)
Adverse maternal outcomes									
Postpartum haemorrhage	15.5%	(199)	15.6%	(47)	15.2%	(75)	15.8%	(77)	0.96 (0.71–1.29)
Transfusion	4.9%	(63)	4.6%	(14)	2.6%	(13)	7.4%	(36)	**0.39 (0.22–0.70)**
Preterm birth (<37 wks)	10.8%	(138)	11.9%	(36)	9.1%	(45)	11.7%	(57)	0.91 (0.63–1.30)
Adverse neonatal outcomes									
Small for gestational age	12.1%	(155)	11.9%	(36)	13.6%	(67)	10.7%	(52)	1.07 (0.75–1.52)
Transfer to NICU/SCN or stillbirth or very low birthweight	21.8%	(280)	25.8%	(78)	18.7%	(92)	22.5%	(110)	0.89 (0.70–1.14)

## Discussion

Among 31,906 singleton pregnancies, 4% of women had Hb <110 g/L and 10% had Hb 140+ g/L at ≤20 weeks of pregnancy. Our results suggest that both low and high Hb at ≤20 weeks are associated with adverse outcomes at the time of birth, in a U-shaped relationship that rises on either side of the lowest risk point at 120–129 g/L. The association between the low Hb and adverse outcomes was relatively stronger than that between high Hb and adverse outcomes. Only transfusion had a linear relationship, with risk increasing with lower Hb and decreasing with higher Hb. The U-shaped relationship between Hb and adverse outcomes that we found has also been shown in a study in Peru in both high and low altitude pregnancies. [22]

Of the women with a low Hb at ≤20 weeks, almost 40% had their Hb restored in the second half of pregnancy. Restoration of Hb did not appear to change risk of PPH, preterm birth, SGA or a composite indicator including transfer to higher care, stillbirth and very low birthweight, but did lower the risk of postpartum transfusion. These data are consistent with a review of trial data suggesting iron supplementation improved Hb levels in pregnant women but did not conclusively improve pregnancy outcomes.[23] The reasons why improvements in Hb do not translate into improved perinatal outcomes require further study. There may be a critical window for the impact of low Hb on outcomes, or the low Hb may be a symptom of an underlying condition that is itself the cause of the poor outcome. Another possibility is that restoring Hb does in fact improve some outcomes, but not those specifically measured in our study.

The higher rate of PPH demonstrated in the low compared with normal Hb groups is in line with previous evidence suggesting anaemia is associated with a higher risk of PPH.[24, 25] Women with high antenatal Hb also had a slightly higher PPH rate than those with normal Hb (although not significantly so), but were less likely to be transfused than those with low Hb, which may have been due to better iron reserves or the treating clinicians being more willing to tolerate blood loss before deciding to transfuse. We also found a significantly higher risk of adverse outcomes such as preterm birth, very low birthweight and transfer to higher care or stillbirth for those with high Hb result, as has been found in other studies,[26, 27] possibly due to inadequate plasma volume expansion, or the impaired response to inflammation and infection,[5, 26] or possibly due to high Hb levels before pregnancy.

Antepartum haemorrhage and abnormal placenta site can cause anaemia and are also associated with adverse pregnancy outcomes.[28, 29] These factors were adjusted for in our analysis, but were also unlikely to have influenced anaemia in the first 20 weeks of pregnancy, as bleeding due to these factors usually occurs later in the pregnancy.

Australian data on the prevalence of anaemia in pregnancy is limited. Our estimate of low Hb (4%) was similar to a 2015 South Australian estimate of women with anaemia in pregnancy (6.6%).[30] International studies have found much higher rates of maternal anaemia with a global estimate of 38% in 2011.[31] The high proportion of women with a history of iron-deficiency anaemia in our population (15%), particularly in the low Hb group, suggest there may have been opportunities to correct low Hb due to iron deficiency before the pregnancy.

We were able to obtain Hb results for a large cohort of pregnant women and examine outcomes by Hb levels at ≤20 weeks gestation. However, limitations of these data were that only Hb results that were manually entered in birth data by midwives or were obtained from in-hospital pathology laboratories (at Royal North Shore) or in-hospital or linked pathology laboratories (at Westmead) were available. This meant there were 13% of pregnant women (n = 4621) who did not have a valid Hb result in the first 20 weeks of pregnancy. These women were broadly similar to those in the final study population, though. Also, we did not know the cause of the low Hb or what measures were taken to restore Hb, and could only infer treatment based on changes in Hb results. From a previous survey, and clinical experience, we assume that a majority of women were taking supplemental iron, either as part of a multivitamin or in an iron-only supplement,[32] but without information on which supplements, and how much iron they contained, collected in the database, we were unable to examine how this impacted on Hb or outcomes. The country of birth results suggest that some thalassaemia/sickle cell anaemia cases may have been missed, as these conditions are more common in Africa and the Middle East, where the low Hb women were more likely to be born.

## Conclusions

We found that women with low Hb in the first 20 weeks of their pregnancy were more likely to have a PPH and blood transfusion after the birth than those with Hb of 120–129 g/L. Those with high and low Hb had increased risk of preterm birth, very low birthweight and a composite outcomes of transfer to higher care or stillbirth compared with those at 120–129 g/L. Restoring the Hb during the pregnancy decreased risk of postpartum transfusion, but did not appear to impact the risk of other adverse outcomes measured. Further research is needed to determine why low and high Hb in the first 20 weeks is associated with poorer outcomes and whether these poor outcomes can be prevented before or during the pregnancy.

## Supporting information

S1 Table Detailed codes. (DOCX)

S2 Table Comparison between study population and those with missing Hb in first 20 weeks. (DOCX)

## References

[1] Patterson J, Roberts C, Bowen J, Irving D, Isbister J, Morris J, et al Obstetric blood transfusion during pregnancy, birth and the postnatal period: a population based study. Obstet Gynecol. 2014;123(1):126–33. 10.1097/AOG.0000000000000054 24463672

[2] Knight M, Callaghan WM, Berg C, Alexander S, Bouvier-Colle MH, Ford JB, et al Trends in postpartum hemorrhage in high resource countries: a review and recommendations from the International Postpartum Hemorrhage Collaborative Group. BMC Pregnancy Childbirth. 2009;9:55 10.1186/1471-2393-9-55 19943928 PMC2790440

[3] Ford JB, Roberts CL, Simpson JM, Vaughan J, Cameron CA. Increased postpartum hemorrhage rates in Australia. Int J Gynaecol Obstet. 2007;98(3):237–43. 10.1016/j.ijgo.2007.03.011 17482190

[4] Muñoz M, Peña-Rosas JP, Robinson S, Milman N, Holzgreve W, Breymann C, et al Patient blood management in obstetrics: Management of anaemia and haematinic deficiencies in pregnancy and in the post-partum period: NATA consensus statement. Transfus Med. 2018;28(1):22–39. 10.1111/tme.12443 28722245

[5] Milman N. Iron and pregnancy—A delicate balance. Annals of Hematology. 2006;85(9):559–65. 10.1007/s00277-006-0108-2 16691399

[6] Milman N. Prepartum anaemia: prevention and treatment. Annals of Hematology. 2008;87(12):949–59. 10.1007/s00277-008-0518-4 18641987

[7] National Blood Authority. Patient Blood Management Guideline: Module 5 –Obstetrics and Maternity. Canberra: National Blood Authority, 2015.

[8] World Health Organization. Haemoglobin concentrations for the diagnosis of anaemia and assessment of severity. Geneva: WHO, 2011 Document No.: WHO/NMH/NHD/MNM/11.1.

[9] Centers for Disease Control and Prevention. Recommendations to prevent and control iron deficiency in the United States. Atlanta, GA: CDC; 1998 [cited 2019 Jan 15]. Available from: https://www.cdc.gov/Mmwr/preview/mmwrhtml/00051880.htm.

[10] Drukker L, Hants Y, Farkash R, Ruchlemer R, Samueloff A, Grisaru-Granovsky S. Iron deficiency anemia at admission for labor and delivery is associated with an increased risk for Cesarean section and adverse maternal and neonatal outcomes. Transfusion. 2015;55(12):2799–806. 10.1111/trf.13252 26246160

[11] Allen LH. Anemia and iron deficiency: effects on pregnancy outcome. Am J Clin Nutr. 2000;71(5):1280–4. 10.1093/ajcn/71.5.1280s 10799402

[12] Haider BA, Olofin I, Wang M, Spiegelman D, Ezzati M, Fawzi WW. Anaemia, prenatal iron use, and risk of adverse pregnancy outcomes: systematic review and meta-analysis. BMJ. 2013;346:f3443 10.1136/bmj.f3443 23794316 PMC3689887

[13] Murphy JF, Newcombe RG, O'Riordan J, Coles EC, Pearson JF. Relation of haemoglobin levels in first and second trimesters to outcome of pregnancy. Lancet. 1986;327(8488):992–5. 10.1016/s0140-6736(86)91269-9 2871331

[14] Department of Health. Clinical Practice Guidelines: Pregnancy Care. Canberra, Australia: Australian Government Department of Health, 2018.

[15] Royal Australian and New Zealand College of Obstetricians and Gynaecologists. Statement C-Obs 25: Vitamin and Mineral Supplementation and Pregnancy. Melbourne: RANZCOG, 2014 Document No.: C-Obs 25.

[16] Isbister JP. The three-pillar matrix of patient blood management—An overview. Best Pract Res Clin Anaesthesiol. 2013;27(1):69–84. 10.1016/j.bpa.2013.02.002 23590917

[17] National Centre for Classification in Health. The International Statistical Classification of Diseases and Related Health Problems, Tenth Revision, Australian Modification (ICD-10-AM), Australian Classification of Health Interventions (ACHI) and Australian Coding Standards (ACS), Seventh Edition. Sydney: National Centre for Classification in Health, 2010.

[18] SNOMED International. What is SNOMED CT? 2018 [cited 2018 Aug 21]. Available from: https://www.snomed.org/snomed-ct/what-is-snomed-ct.

[19] Austin PC. Balance diagnostics for comparing the distribution of baseline covariates between treatment groups in propensity-score matched samples. Statistics in Medicine. 2009;28(25):3083–107. 10.1002/sim.3697 19757444 PMC3472075

[20] Dobbins TA, Sullivan EA, Roberts CL, Simpson JM. Australian national birthweight percentiles by sex and gestational age, 1998–2007. Med J Aust. 2012;197(5):291–4. 10.5694/mja11.11331 22938128

[21] Australian Bureau of Statistics. 2033.0.55.001—Census of Population and Housing: Socio-Economic Indexes for Areas (SEIFA), Australia, 2011 2011 [cited 2018 Jun 15]. Available from: http://www.abs.gov.au/AUSSTATS/abs@.nsf/allprimarymainfeatures/8C5F5BB699A0921CCA258259000BA619?opendocument.

[22] Gonzales GF, Steenland K, Tapia V. Maternal hemoglobin level and fetal outcome at low and high altitudes. American Journal of Physiology-Regulatory, Integrative and Comparative Physiology. 2009;297(5):R1477–R85. 10.1152/ajpregu.00275.2009 19741055 PMC2777782

[23] Peña‐Rosas JP, De‐Regil LM, Garcia‐Casal MN, Dowswell T. Daily oral iron supplementation during pregnancy. Cochrane Database Syst Rev. 2015;(7). 10.1002/14651858.CD004736.pub5 PMC8918165 26198451

[24] Owiredu WKBA, Osakunor DNM, Turpin CA, Owusu-Afriyie O. Laboratory prediction of primary postpartum haemorrhage: A comparative cohort study. BMC Pregnancy Childbirth. 2016;16(1). 10.1186/s12884-016-0805-3 PMC4727344 26810108

[25] Kavle JA, Stoltzfus RJ, Witter F, Tielsch JM, Khalfan SS, Caulfield LE. Association between anaemia during pregnancy and blood loss at and after delivery among women with vaginal births in Pemba Island, Zanzibar, Tanzania. J Health Popul Nutr. 2008;26(2):232–40. 18686556 PMC2740668

[26] Dewey KG, Oaks BM. U-shaped curve for risk associated with maternal hemoglobin, iron status, or iron supplementation. Am J Clin Nutr. 2017;106:1694S–702S. 10.3945/ajcn.117.156075 29070565 PMC5701708

[27] Gonzales GF, Tapia V, Fort AL. Maternal and Perinatal Outcomes in Second Hemoglobin Measurement in Nonanemic Women at First Booking: Effect of Altitude of Residence in Peru. ISRN Obstetrics and Gynecology. 2012;2012:7. 10.5402/2012/368571 PMC3345214 22577573

[28] Sinha P, Kuruba N. Ante-partum haemorrhage: An update. J Obstet Gynaecol. 2008;28(4):377–81. 10.1080/01443610802091487 18604667

[29] Vahanian SA, Lavery JA, Ananth CV, Vintzileos A. Placental implantation abnormalities and risk of preterm delivery: A systematic review and metaanalysis. Am J Obstet Gynecol. 2015;213(4):S78–S90. 26428506 10.1016/j.ajog.2015.05.058

[30] Scheil W, Jolly K, Scott J, Catcheside B, Sage L, Kennare R. Pregnancy Outcome in South Australia 2015. Adelaide: Pregnancy Outcome Unit, SA Health, Government of South Australia, 2017.

[31] Stevens GA, Finucane MM, De-Regil LM, Paciorek CJ, Flaxman SR, Branca F, et al Global, regional, and national trends in haemoglobin concentration and prevalence of total and severe anaemia in children and pregnant and non-pregnant women for 1995–2011: A systematic analysis of population-representative data. Lancet Global Health. 2013;1(1):E16–E25. 10.1016/S2214-109X(13)70001-9 25103581 PMC4547326

[32] Chatterjee R, Shand A, Nassar N, Walls M, Khambalia AZ. Iron supplement use in pregnancy—Are the right women taking the right amount? Clin Nutr. 2016;35(3):741–7. 10.1016/j.clnu.2015.05.014 26070630

