# Supplementary material for: The association between haemoglobin levels in the first 20 weeks of pregnancy and pregnancy outcomes
Source: PLoS One. 2019 Nov 13;14(11):e0225123. doi: 10.1371/journal.pone.0225123 (PMC6853312; doi:10.1371/journal.pone.0225123)
Supplement: S1 Table — (DOCX) [file pone.0225123.s001.docx]

**S1 Table. Detailed codes.**

| Outcomes |  |
| --- | --- |
| Postpartum haemorrhage | ICD-10-AM codes in the eMR after birth or in first 6 weeks postnatally:  O72.0- Third-stage haemorrhage  O72.1 - Other immediate postpartum haemorrhage  O72.2 - Delayed and secondary postpartum haemorrhage  O72.3 - Postpartum coagulation defects  OR  ObstetriX variables:  Blood volume at delivery ≥500 mL  OR  Cumulative blood volume at discharge ≥500 mL |
| Transfusion at birth or postnatally | Transfusion in birth admission (after birth, not before) or in first 6 weeks postnatally.  ICD-10-AM codes:  Z51.3 - Blood transfusion wo reported diagnosis  ACHI codes:  13706-01 whole blood transfusion  13706-02 packed cells transfusion  13706-03 platelet transfusion  92061-00 Administration of coagulation factors  92062-00 Administration of other serum  92206-00 exchange transfusion  SNOMED codes:  413852010 massive blood transfusion  428230014 blood transfusion |
| Neonatal transfer to SCN/NICU | ObstetriX variables  Neonatal admission to special care nursery or neonatal intensive care at birth  Admitted to NICU - birth defect  Admitted to NICU - other reason  Admitted to SCN - birth defect  Admitted to SCN - other reason  VS  Not admitted to NICU or SCN  Removing: Missing  AND/OR  Neonatal admission to special care nursery or neonatal intensive care at birth OR after birth  Admitted to NICU - birth defect  Admitted to NICU - other reason  Admitted to SCN - birth defect  Admitted to SCN - other reason  VS  Not admitted to NICU or SCN |
| Small for gestational age <10% | Birth weight (ObstetriX) and gestational age (ObstetriX) compared with gestational age-specific ranges (female ranges applied to all infants as infant sex was not available)^19^ |
