# Supplementary material for: The association between haemoglobin levels in the first 20 weeks of pregnancy and pregnancy outcomes
Source: PLoS One. 2019 Nov 13;14(11):e0225123. doi: 10.1371/journal.pone.0225123 (PMC6853312; doi:10.1371/journal.pone.0225123)
Supplement: S2 Table — (DOCX) [file pone.0225123.s002.docx]

**S2 Table. Comparison between study population and those with missing Hb in first 20 weeks.**

|  | **Total** | | **Missing Hb in first** | | **Standardised** |
| --- | --- | --- | --- | --- | --- |
|  |  | | **20 weeks** | | **Difference** |
|  | **n=31,906** | | **n=4621** | |  |
|  | **Col % (n)** | | **Col % (n)** | |  |
| ***Maternal characteristic*** |  |  |  |  |  |
| **Age group**‡ |  |  |  |  |  |
| <20 | 1.1% | (355) | 2.6% | (121) | **0.11** |
| 20-34 | 76.4% | (24362) | 74.1% | (3424) | -0.05 |
| 35+ | 22.5% | (7189) | 23.3% | (1076) | 0.02 |
| **Country of birth**‡ |  |  |  |  |  |
| Australia | 35.2% | (11227) | 39.8% | (1840) | 0.10 |
| Oceania (rest) | 3.6% | (1134) | 6.3% | (291) | **0.12** |
| Europe | 6.8% | (2158) | 4.9% | (228) | -0.08 |
| Africa and the Middle East | 10.2% | (3253) | 10.8% | (500) | 0.02 |
| South- and North-East Asia | 19.8% | (6322) | 21.7% | (1002) | 0.05 |
| Southern Asia | 19.8% | (6332) | 13.3% | (615) | **-0.18** |
| Central Asia | 2.3% | (721) | 1.7% | (79) | -0.04 |
| Americas | 2.4% | (759) | 1.4% | (66) | -0.07 |
| **Socio-economic status quintile**‡ |  |  |  |  |  |
| 1 - most disadvantaged | 21.3% | (6811) | 23.6% | (1091) | 0.06 |
| 2 | 4.4% | (1406) | 6.3% | (292) | 0.08 |
| 3 | 22.6% | (7206) | 21.5% | (993) | -0.03 |
| 4 | 19.3% | (6147) | 18.7% | (863) | -0.02 |
| 5 - most advantaged | 32.4% | (10336) | 29.9% | (1382) | -0.05 |
| ***Previous history*** |  |  |  |  |  |
| **Parity**† |  |  |  |  |  |
| No previous births | 47.4% | (15117) | 46.2% | (2137) | -0.02 |
| 1 previous birth | 34.5% | (10999) | 31.4% | (1452) | -0.07 |
| 2+ previous births | 18.1% | (5790) | 22.3% | (1032) | **0.10** |
| **Medical history** |  |  |  |  |  |
| Pre-existing diabetes† | 0.9% | (287) | 1.0% | (46) | 0.01 |
| Previous gestational diabetes† | 4.3% | (1358) | 4.0% | (187) | -0.02 |
| Pre-existing hypertension | 6.3% | (1997) | 7.1% | (330) | 0.03 |
| Previous major uterine surgery† | 16.4% | (5229) | 17.6% | (813) | 0.03 |
| **Previous history of anaemia†** |  |  |  |  |  |
| Iron-deficiency | 14.9% | (4764) | 16.0% | (740) | 0.03 |
| B12/folate deficiency | 0.3% | (97) | 1.2% | (54) | **0.10** |
| ***Current pregnancy risks*** |  |  |  |  |  |
| Smoked during pregnancy‡ | 2.8% | (892) | 5.7% | (265) | **0.14** |
| Gestational diabetes‡ | 11.6% | (3688) | 10.5% | (483) | -0.04 |
| Pregnancy hypertension‡ | 5.0% | (1592) | 6.2% | (287) | 0.05 |
| Abnormal placental site‡ | 1.4% | (437) | 1.8% | (83) | 0.03 |
| Antepartum haemorrhage‡ | 3.9% | (1260) | 4.4% | (204) | 0.03 |
| **Body Mass Index**† |  |  |  |  |  |
| <18.5 | 5.9% | (1879) | 6.3% | (291) | 0.02 |
| 18.5-24.99 | 59.6% | (19023) | 55.5% | (2563) | -0.08 |
| 25+ | 34.5% | (11004) | 38.2% | (1765) | 0.08 |
| ***Birth factors*** |  |  |  |  |  |
| **Labour onset** |  |  |  |  |  |
| Spontaneous | 52.5% | (16739) | 51.1% | (2360) | -0.03 |
| Induction | 30.6% | (9766) | 28.1% | (1298) | -0.05 |
| Pre-labour caesarean | 16.9% | (5401) | 20.8% | (963) | 0.10 |
| **Mode of birth** |  |  |  |  |  |
| Vaginal unassisted | 57.2% | (18260) | 56.4% | (2604) | -0.02 |
| Vaginal instrumental – forceps | 7.8% | (2480) | 7.1% | (328) | -0.03 |
| Vaginal instrumental – vacuum | 4.9% | (1571) | 3.7% | (172) | -0.06 |
| Caesarean section | 30.1% | (9595) | 32.8% | (1517) | 0.06 |
|  |  |  |  |  |  |
| **Perineal trauma (for vaginal births only, n=22,311 and n=3104)** | | | | |  |
| None | 29.1% | (6482) | 33.6% | (1044) | 0.10 |
| 1^st^ degree / Other | 30.3% | (6758) | 31.0% | (961) | 0.02 |
| 2^nd^ degree | 36.1% | (8052) | 32.1% | (997) | -0.08 |
| 3^rd^ or 4th degree | 4.6% | (1019) | 3.3% | (102) | -0.07 |
|  |  |  |  |  |  |
