# Supplementary material for: The association between haemoglobin levels in the first 20 weeks of pregnancy and pregnancy outcomes
Source: PLoS One. 2019 Nov 13;14(11):e0225123. doi: 10.1371/journal.pone.0225123 (PMC6853312; doi:10.1371/journal.pone.0225123)
Supplement: Attachment — Submitted filename: R2R.docx [file pone.0225123.s003.docx]

***Editors***

**Response:** We have updated the formatting of the manuscript according to the PLOS One formatting guidelines.

***Reviewer #1***

*The study aimed to assess the association of haemoglobin (Hb) in the first 20 weeks of pregnancy, and restoration of low Hb levels, with pregnancy outcomes in Australia. The study included 31,906 singletons births data obtained from 2011 to 2015 from hospital records. Women with both low and high Hb in the first 20 weeks of pregnancy had higher risks of adverse outcomes than those with normal Hb. Restoring Hb after 20 weeks did not improve most adverse outcome rates but did reduce risk of transfusion.*

*This is an important findings since suggest that women anemic in the first half of pregnancy probably were anemics since before pregnancy. This is suggested form one of the results of the study. This may allow to give treatment only to those women qualified as anemic before they get pregnant.*

*It is interesting the U shape for fetal outcomes. A previous paper demonstrated this U shape in populations living at low and at high altitude (Gonzales et al., 2009) indicating that association could be global.*

**Response:** Thank you for highlighting this previous study. We have added these findings into the discussion.

Page 12, line 216: “The U-shaped relationship between Hb and adverse outcomes that we found has also been shown in a study in Peru in both high and low altitude pregnancies.”

*The absence of improvement after restored Hb at the second half of pregnancy is also important finding suggesting that use of iron supplementation on this period of gestation should be limited. This is in accordance with a previous findings that increase in Hb concentration in a second booking after a normal Hb at first booking resulted in double the risk of small for gestational age (Gonzales et al., 2012).*

**Response:** Thank you for highlighting this study. We have added this study as a reference for the poor outcomes for those with high haemoglobin at Page 13, line 236.

*In realtion to pregnant women with high Hb levels, authors suggest inadequate plasma volume expansion (hemoconcentration) or impaired response to inflammation and infection. Authors should also consider that women may also have high Hb levels before pregnancy.*

**Response:** Thank you for this comment. We have added this as a possibility in the Discussion.

Page 13, line 237: “…or possibly due to high Hb levels before pregnancy.”

*Minor comments:*

*In the Table there is no number of cases for 140+ g/L group. Instead appears a column with percentages. Please check.*

**Response:** Thank you for picking up this error. We have fixed this in the revised manuscript.

***Reviewer #2***

*Randall et al conducted a retrospective cohort study analyzing the association of haemoglobin values in early pregnancy (<20 weeks) with pregnancy outcomes, maternal post-partum haemorrhage and transfusion up to 6 weeks post-partum. Data was collected from two large public hospitals in New South Wales, Australia. An appropriately large group of subjects was included in the analysis - 31,906 singleton pregnancies - and the cohort was split into three groups based on their Hb levels (Hb <110g/L, 110-139g/L and >140g/L) for analysis. Overall, there was a U-shaped association between maternal Hb values prior to 20 weeks gestation and adverse pregnancy outcomes, with the Hb <110g/L group having the greatest percentage of adverse outcomes. Transfusion was the only outcome that displayed a linear relationship with decreased risk with increasing Hb levels. This study supports previous studies demonstrating that low maternal Hb along with high maternal hemoglobin results in increased negative pregnancy outcomes. Importantly, the study found that restoring the Hb during the pregnancy did not improve adverse outcomes except for the risk of postpartum transfusion. This study highlights the need to for further studies to understand why and how low and high Hb are associated with poorer outcomes and how these poor outcomes can be prevented before or during the pregnancy.*

*Questions/Comments:*

*1) Provide explanation for why lowest Hb was selected rather than the most recent Hb at <20 weeks of gestation.*

**Response:** We chose the lowest Hb in the first 20 weeks, as we were interested in the value of Hb that might have triggered treatment, rather than a Hb value that might have been measured after early treatment. We have included further explanation of this in the Methods.

Page 5, line 104: “We chose the lowest Hb in the first 20 weeks as we were interested in the Hb before any treatment.”

*2) It may be helpful to include the range of Hb values for both the low and high Hgb group.*

**Response:** Thank you for this suggestion. We have included the Hb ranges for the low and high Hb groups.

Page 5, line 101-102: “… as well as three broad categories of <110 (range 45-109), 110-139 and 140+ (range 140-159) g/L”.

*3) Table 1. n not provided for >140g/L group, instead average percentage is presented in table.*

**Response:** Thank you for picking up this error. We have fixed this in the revised manuscript.

*4) Could the authors speculate on why restoring Hb during the pregnancy did not improve most of the adverse outcomes?*

**Response:** There are a number of hypotheses as to why this might be the case. There might be critical windows for the impact of low Hb, and as such, restoring the Hb after this critical time may not be able to reverse any ‘damage’ already done. It may be because the low Hb is a symptom of an underlying condition, and this condition, not the Hb itself, is the cause of the poor outcomes. Or perhaps restoring the Hb may be improving health outcomes, but not the outcomes that are being measured in this study. We have included further discussion of this in the Discussion section.

Page 13, line 224: “The reasons why improvements in Hb do not translate into improved perinatal outcomes require further study. There may be a critical window for the impact of low Hb on outcomes, or the low Hb may be a symptom of an underlying condition that is itself the cause of the poor outcome. Another possibility is that restoring Hb does in fact improve some outcomes, but not those specifically measured in our study.”
